# Clinical characteristics and management experience of schwannoma in extremities: Lessons learned from a 10-year retrospective study

**DOI:** 10.3389/fneur.2022.1083896

**Published:** 2022-12-15

**Authors:** Haiying Zhou, Chengjun Yao, Yanzhao Dong, Ahmad Alhaskawi, Zewei Wang, Jingtian Lai, Sohaib Hasan Abdullah Ezzi, Vishnu Goutham Kota, Mohamed Hasan Abdulla Hasan Abdulla, Hui Lu

**Affiliations:** ^1^Department of Orthopedics, The First Affiliated Hospital, Zhejiang University School of Medicine, Hangzhou, China; ^2^Department of Clinical Medicine, Zhejiang University School of Medicine, Hangzhou, China; ^3^Department of Orthopaedics of the Third Xiangya Hospital, Central South University, Changsha, China; ^4^Alibaba-Zhejiang University Joint Research Center of Future Digital Healthcare, Zhejiang University, Hangzhou, China

**Keywords:** schwannoma, extremity, retrospective study, diagnosis, microsurgery, prognosis

## Abstract

**Introduction:**

Schwannomas are the most common neoplastic lesions of the peripheral nerves when growing on the extremities, they usually have adverse effects on patients due to the exposed and functional nature of the region.

**Methods:**

In the present single-center retrospective study, we included all patients with pathologically confirmed schwannoma located in extremities between 2011 and 2021 totaling 183 patients. Data on gender, age, duration history, clinical presentation, occurrence region, nerve affiliation, imaging data, modus operation, mass volume, immunohistochemistry, postoperative neurological function, and recurrence were collected.

**Results:**

As in previous studies, patients were predominantly middle-aged with a mean age of 49.5, without gender preference and a male-to-female ratio of 1.2:1. Most patients are first seen for this disease, and only five of them are recurrent. The majority presented with an isolated (91.26%), asymptomatic (37.7%) mass, with tenderness (34.97%) being the second frequent complaint. 60% of lesions occurred in the upper extremity, more commonly on the left side (55.26%) than the right. The average duration of onset was 47.50 months. MRI is more sensitive for neurogenic tumors than ultrasound, as it owns 78.93% correct. In immunohistochemistry, the top three markers for positive labeling schwannoma are S-100 (98.95%), Ki67 (98.68%) and β-Catenin. 98.36% of patients underwent complete resection of the lesion, of which 14.44% required partial sacrifice of the nerve fibers. Thanks to the application of intraoperative peripheral nerve microscopic operation, only 6 patients showed symptoms of postoperative nerve injury, and 3 of them received second surgery. Intraoperative microscopic manipulation, preservation of the main nerve, and the need for reconstruction of the affected nerve fibers are some of the points worth noting.

**Discussion:**

In summary, the possibility of schwannoma should not be overlooked in the identification of masses that occur in the upper extremities of the middle-aged population. Preoperative ultrasound and MR are useful for determining the nature of the mass, and S100, Ki67, and β-Catenin are sensitive to it. Surgical resection can achieve satisfying functional results and a low risk of nerve injury.

## Introduction

Schwannoma, also known as neurilemmoma, is a rare disease but is also the most common type of peripheral nerve sheath tumor (1). It consists solely of benign tumorigenic Schwann cells, therefore, often has an intact capsule. Schwannoma can occur anywhere in the body where nerve fibers are located, with the extremities being one of the most common sites, accounting for 32.6% of occurrences (2, 3). Although they often appear as isolated, painless, slow-growing masses, the abundant nerve peripheral occupation region and variable size of the masses can sometimes present with physical symptoms such as nerve deficits or pain. Complete surgical resection is often considered sequelae-free, but in some research, a poor prognosis of neurological deficits has been reported (4–7).

Because of its rarity and rich clinical presentation, patients with schwannoma consult diverse departments, resulting in delays and missed diagnoses, as well as different treatment options. Meanwhile, only a small portion of the literature has meticulously investigated schwannoma in extremities, and data on masses originating from the terminal branches are often neglected, resulting in rare relevant diagnoses and treatment experiences (8–10). Therefore, this study collected clinical data from 183 patients with schwannoma at the extremity treated at a large general hospital in the last decade, and to our knowledge, this is the largest retrospective study of schwannomas of the extremities. In this study, we summarize the clinical features of schwannoma and explore the key points of clinical diagnosis and treatment of this disease.

## Materials and methods

We retrospectively reviewed all medical records of the patients who were hospitalized in our hospital from 2011 to 2021, received surgical treatment, and were finally confirmed as schwannoma by pathology. The tumors must be located at the extremities, not the trunk or head and neck. Patients with indefinite pathological types such as spindle cell soft tissue tumors, traumatic neuroma, and neurogenic tumors were excluded. Data included clinical, surgical, and radiological findings. All patients were followed up for 1–10 years. Most patients were discharged after 1 year of follow-up due to no discomfort.

The preoperative characteristics containing gender, age, time of the first detection of the mass, location, size of the mass, major complaints, previous medical experience, and physical examination discovery were collected. It is important to note that the physical examination is often incomplete as the judgment of such disease may vary between different medical history collectors. Patient imaging data, including ultrasound, CT, X-rays, and MRI, were also recorded in detail. However, as X-rays and CT were used less frequently on such patients and the data were so sketchy, we removed them from the analysis. The results of ultrasound and MRI, on the other hand, were categorized into three types based on the diagnostic information they ultimately provided, namely negative, where the mass could not be detected; positive, where the mass was detected but the provisional diagnosis did not consider a neurogenic tumor; and correct, where the diagnosis considered the mass to be a neurogenic tumor.

All masses were surgically excised, and the surgical approach and intraoperative relationship between the mass and surrounding tissues, the size and number of mass, and the involved nerves were tracked. During the surgery, we found that the peeled masses were mostly ellipsoidal or fusiform, so the volume of the mass was calculated using the formula of an ellipsoid: Volume (cm^3^) = (V = 4/3 × π × a × b × c; where a, b, and c represent the three semi-axes). The results of postoperative pathology and immunohistochemical markers were also collected as the gold standard for diagnosis. Clinical follow-up started at 2 weeks postoperatively and then continued for 1, 3 months, or even longer; subsequently, every patient should be followed over 1 year. At each follow-up visit, clinical outcomes, such as the healing of the patient's incision, improvement in chief complaints, and limb sensation, and motor function were assessed and recorded.

Quantitative variables were described using means and standard deviations (SD) and compared using *t*-tests or non-parametric tests, when the data are not a normal distribution, such as the tumor volume and medical history time. Qualitative data were described using percentages or numbers and used one-way ANOVA or Chi-square test to analyze the difference. The level of significance was set to *p* < 0.05. All analyses were done in SPSS 23.0.

## Results

### General characteristics of patients and tumors

Data were collected from a total of 330 patients with a pathological diagnosis of schwannomas; of which, 147 patients were screened out due to their inadequate medical records, insufficient follow-up time, and incorrect tumor site. Eventually, 183 patients and 190 sites of masses were included in the study, with five patients having two different sites of schwannoma and one patient having three different sites of masses. The mean age of the patients was 49.5 ± 14.5 years, ranging from 7 to 80 years, and male patients were slightly more than female patients (100:83) (Table 1). Five of the patients had a history of schwannoma resection at other hospitals before this hospitalization, ranging from 1 to more than 10 years.

**Table 1:** Summary of clinical characteristics of patients and masses.

**Clinical characteristics**		**No. of patients of masses**
Age (years)	≤20	2
49.5 ± 14.5	21–30	34
	31–40	34
	41–50	35
	51–60	49
	61–70	22
	>70	7
Gender	Male	100
	Female	83
Duration time (months)	< 12	47
47.97 ± 57.64	12–60	102
	>60	41
Location	Upper / Lower	114/76
	Flexor / Extensor	115/75
	Left / Right	105/85
Nerve	Main trunk	72
	Branches	118
Volume (cm^3^)	≤64.45 (d = 5 cm)	136
9.44 ± 28.62	>64.45	4
Radiology	Ultrasound	68/13
(right/wrong)	MRI	95/20

In total, 115 masses were found on the flexor side of the body, while 75 masses were found on the extensor side. The majority of masses occurred in the upper extremity (114 cases in total), at the same time, more schwannomas were found in the left side of the extremity (105 cases). Further, according to its soft tissue occupancy, the extremity sites were subdivided into the digit/toe area (digit); the metacarpal region of the hand and foot (palmoplantar); the articular area (joint); and the long bone area of the extremity (limb), with the limb area having the most tumors (93 cases) and the digital area the least (25 cases) (Table 1).

**Figure 1:**
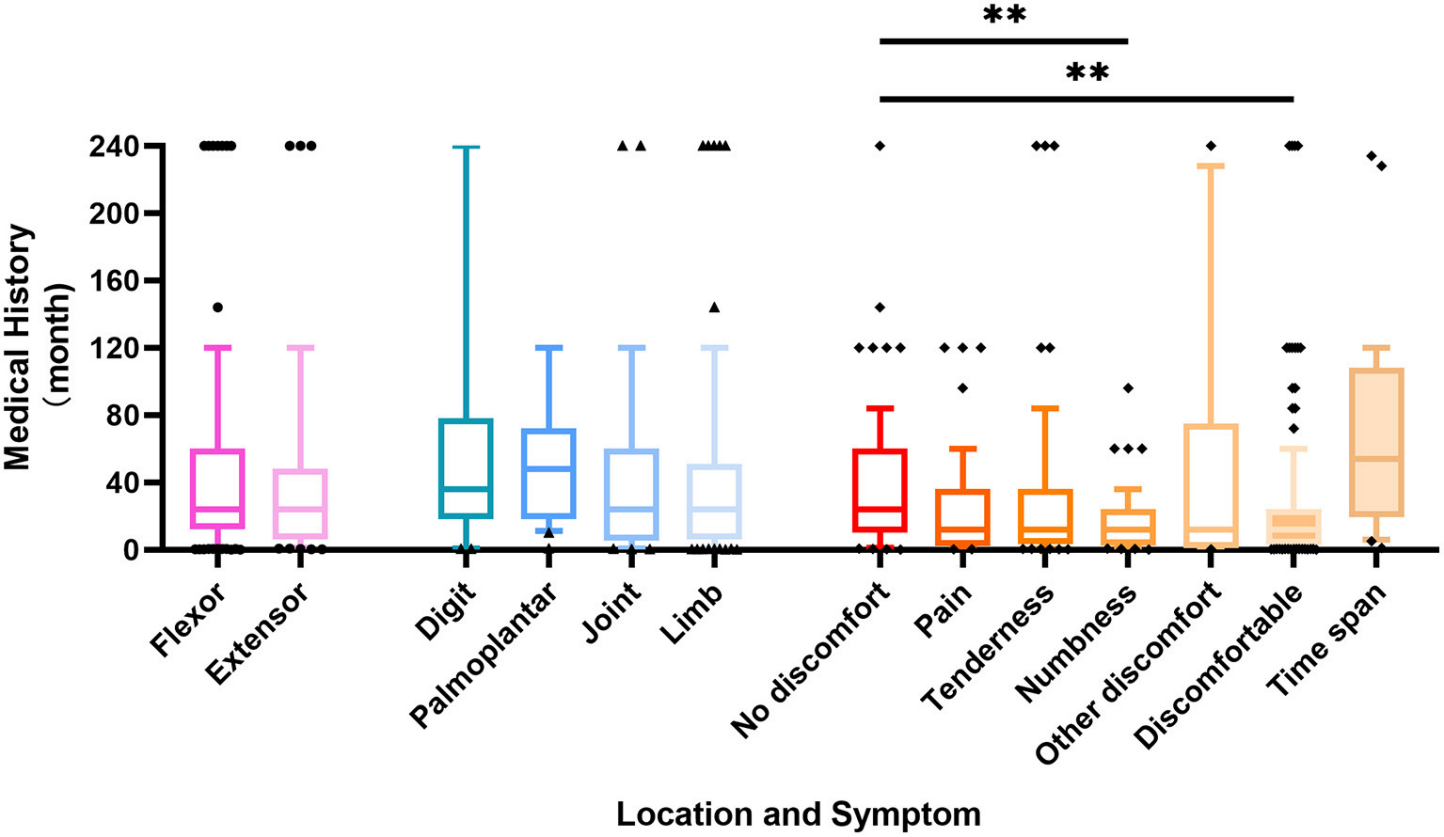
Box plot representing the correlation between patient history time and schwannomas location and symptoms. *p*-value ** < 0.01.

The duration of the disease varies from 7 days to 20 years, and the mean time span between the discovery of the tumor and hospitalization was 47.50 ± 57.40 months. Schwannomas located on the extensor side seeking medical help are at an average of 42.44 months after discovery and the limb mass required an average of 42.57 months. There were no significant differences in the time to seek medical help between sites. Based on the patient's history and a brief physical examination, we classified the patient's complaints into five major categories, namely, no discomfort, pain, tenderness, numbness, and other discomforts (including impaired mobility, decreased muscle strength, pruritus, and interference with nail growth). The majority of patients seek medical treatment solely for masses without any discomfort, accounting for about 37.7%, and their average duration time was as long as 39.08 months. In contrast, patients with numbness had the shortest time to seek medical help with a mean of 15.54 months, significantly shorter than those without complaints. Over 1 year after the discovery of an asymptomatic mass, 87.5% of patients from the discomfort group presented with complaints of discomfort (Figure 1).

### Information of tumors

A total of 159 patients provided preoperative localized imaging of the mass, either at our hospital or the outside, of whom 102 underwent ultrasound and 119 performed MRI, including enhanced MRI. Ultrasound had a sensitivity of 66.67% for neurogenic masses, while 79.83% for MRI is significantly higher (Table 1).

**Figure 2:**
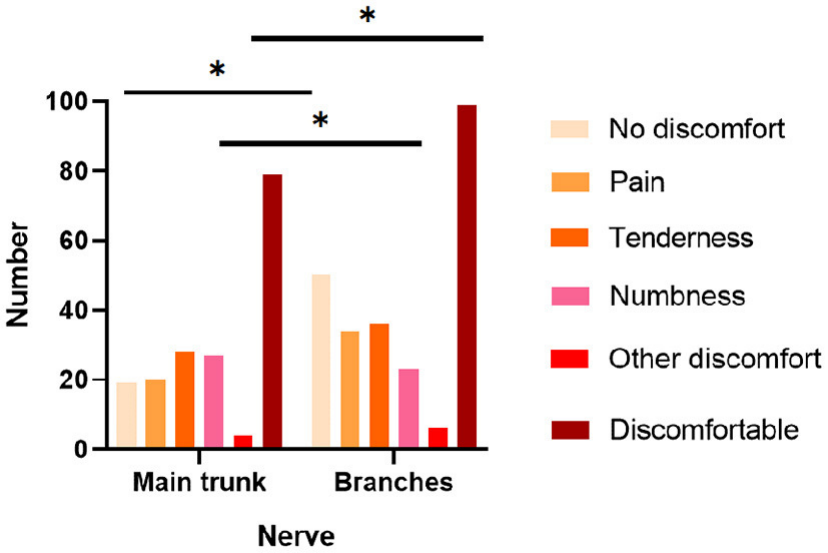
Relationship between the nerve of origin of the mass and the symptoms. *p*-value * < 0.05.

Combining the intraoperative findings with the physical examination and preoperative image, we clarified the nerve to which the patient's schwannomas belonged. Still, 25 sites of masses could not be accurately localized. According to the results, we classified the affected nerves as digital nerve (31 cases), radial nerve (9 cases), ulnar nerve (22 cases), median nerve (24 cases), sciatic nerve (7 cases), common peroneal nerve (18 cases), tibial nerve (18 cases), and other nerves (62 cases) (Table 2). Incidentally, one of the patients had multiple masses in the wrist, one in the ulnar nerve and one in the median nerve. On the other hand, based on whether they originated from the nerve trunk, masses were divided into the main trunks group (72 cases) and the branches group (118 cases). The branches were mostly seen with no complaints, accounting for 33.56%, which was significantly higher than that of the main trunk. In contrast, the nerve trunk was more often accompanied by a numbness (27.55%) and pressure pain (28.57%), and the incidence of numbness was significantly higher than that of the branches (Figure 2). The digital nerve, radial nerve, and sciatic nerve were most often seen without significant discomfort, while the ulnar and common peroneal nerves were often seen with tenderness, the median nerve was frequently seen with pain, and the tibial nerve was seen with more variable complaints (Table 2).

**Table 2:** Detailed information of the single site Schwannomas (*n* = 190).

**Location and affiliated nerve**		**Symptom**					**Surgical treatment**		**Postoperative**	
		**No discomfort**	**Pain**	**Tenderness**	**Numbness**	**Other discomfort**	**Partial resection**	**Complete resection (Sacrifice nerve / neurolysis)**	**Discomfort**	**Reoperation**
Upper extremity	Digital nerve	14	5	9	3	1	0	30 (3/4)	0	0
	Radial nerve	2	4	3	2	2	0	9 (2/1)	1	1
	Ulnar nerve	5	7	9	7	0	1	21 (4/3)	1	1
	Median nerve	10	4	6	8	0	1	23 (2/4)	2	1 (recurrence)
	Other nerves	12	10	11	10	1	0	30 (3/4)	1	0
Lower extremity	Digital nerve	1	0	0	0	0	0	1 (0/1)	0	0
	Sciatic nerve	4	1	1	2	1	0	7 (0/2)	0	0
	Common peroneal nerve	4	7	11	6	2	2	16 (6/0)	0	0
	Tibial nerve	3	8	8	8	1	0	18 (3/3)	2	1
	Other nerves	14	10	8	5	2	0	32 (1/3)	0	0

**Figure 3:**
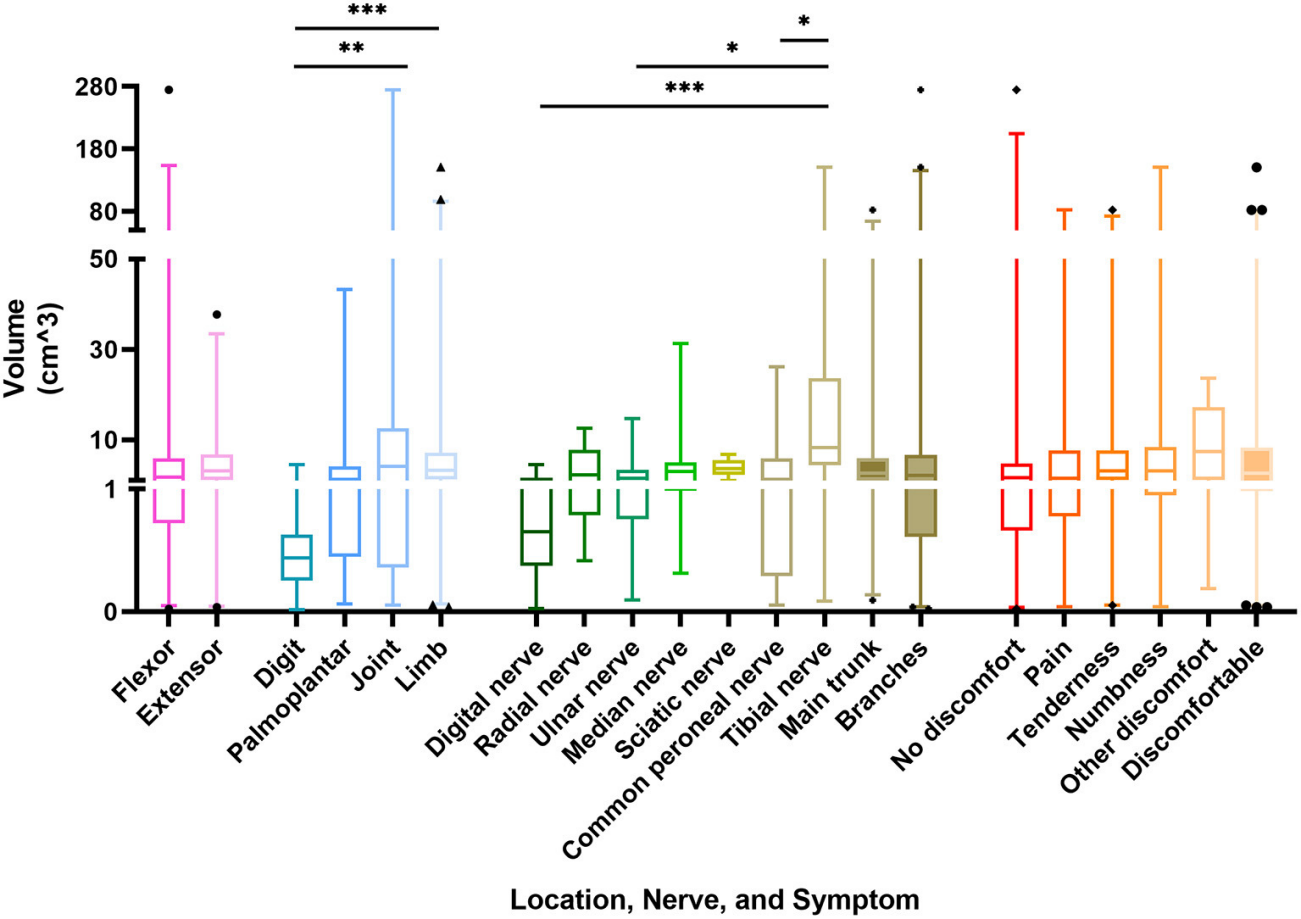
Box plot representing the correlation between the volume of schwannomas and its location, nerve of origin, and symptoms. *p*-value * < 0.05, ** < 0.01, *** < 0.005.

The volume data of the schwannomas are best obtained from the pathologist's report, followed by the surgical records and imaging reports, and the data obtained from the physical examination is generally not considered. However, due to the different labeling habits of physicians, some masses lacked data or only possessed two-dimensional data, which made the volume of the masses difficult to estimate. Consequently, a total of 140 masses obtained accurate volume, which varies from 0.025 to 274.89 cm^3^. The mean volume of the schwannomas was 9.44 ± 28.62 cm^3^. The masses on the extensor side were relatively small at 5.94 cm^3^ and, not surprisingly, the masses on the digital region were the smallest at 0.78 cm^3^, significantly smaller than the masses on the joint and limb. There was no significant difference in the size of schwannomas located in the main trunk or the branches of nerves. While talking about the exact origin nerve, the masses from the tibial nerve were significantly larger than the rest of the groups, with a mean volume of 29.36 cm^3^. Meanwhile, there was also no significant difference in the volume of the masses among symptom groups (Figure 3).

### Intraoperative findings and prognosis

**Figure 4:**
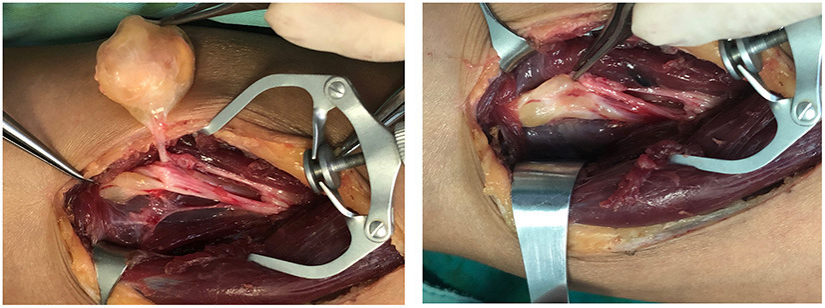
Multiple schwannomas on the median nerve.

Intraoperatively, multiple masses were found in a single site in 10 patients, and in three cases, the nerve showed bead-like masses, and in most cases, 12 small masses were stripped from a single nerve (Figure 4; Supplementary Figure 1). A total of 187 sites of masses were completely excised, and among them, 50 masses affected their surrounding tissues, of which 26 masses sacrificed the encroached nerve fibers or the distal nerves and 24 masses underwent neurolysis and tenolysis.

During the postoperative follow-up, we did not consider the patient's short-term, about 14 days, postoperative symptoms as sequelae of the surgery, given that this could be a transient manifestation of postoperative tissue edema and not due to the nerve injury; also, studies have confirmed an extremely high rate of spontaneous remission of short-term postoperative sensory loss (9). In this definition, 96.72% of patients achieved a complete functional improvement of the extremities without any sequelae after the sophisticated microsurgery. Of note, one patient had a recurrence of the mass 2 years after surgery and underwent surgical resection again. A total of six patients had discomforts such as pain and swelling in the operated area or even limitation of limb movement during the postoperative follow-up period and three of them received further surgical treatment (Supplementary Figure 2; Table 2).

### Immunohistochemical characteristics

During the data collecting process, we found that many patients had an initial pathological diagnosis of spindle cell soft tissue tumor, which was later confirmed by immunohistochemistry as schwannomas, while a part of patients, who did not undergo follow-up to reimbursement for immunohistochemistry due to good postoperative functional recovery, did not have further clarification of the tumor type. A total of 95 masses underwent further immunohistochemical analysis, with the highest positive rate of 98.95% for S-100, followed by 98.68% for Ki-67, and 89.66% for β-Catenin (Table 3).

**Table 3:** Number of positive (+) and negative (-) staining cases of different immunohistochemical markers.

	**(+)**	**(+) / (-)**	**(-)**
S-100	94	0	1
Ki-67	75	0	1
SMA	5	0	66
CD34	25	0	46
Desmin	0	1	65
CD117	2	1	47
CK(pan)	32	0	32
β-Catenin	26	0	6
DOG-1	0	0	26
EMA	0	0	15
HMB45	1	0	13
SOX10	12	0	0
Staining cases < 10	Nestin (4,0,1); SDHB (4,0,0); Vimentin (3,0,0); GFAP (1,0,2); H3K27Me3 (1,0,0); Fli-1 (1,0,0)	/	STAT6 (0,0,6); ALk (0,0,4); Melan A (0,0,2); NF (0,0,2); CD68 (0,0,1); Calponin (CP) (0,0,1); Syn (0,0,1); CgA (0,0,1); ERG (0,0,1); MUC-4 (0,0,1); Myogenin (0,0,1); MyoD1 (0,0,1)

## Discussion

Schwannomas are benign tumors of the peripheral nerves commonly found in the percutaneous nerves and the head, neck, or extremities. Due to their rarity and the diversity of symptoms, only a few studies have been conducted to analyze and summarize the clinical features and treatment points of schwannoma in detail. Furthermore, as far as the author knows, this study investigated the largest number of patients with schwannomas in the extremities (8, 11).

### Epidemiological information of schwannomas

**Figure 5:**
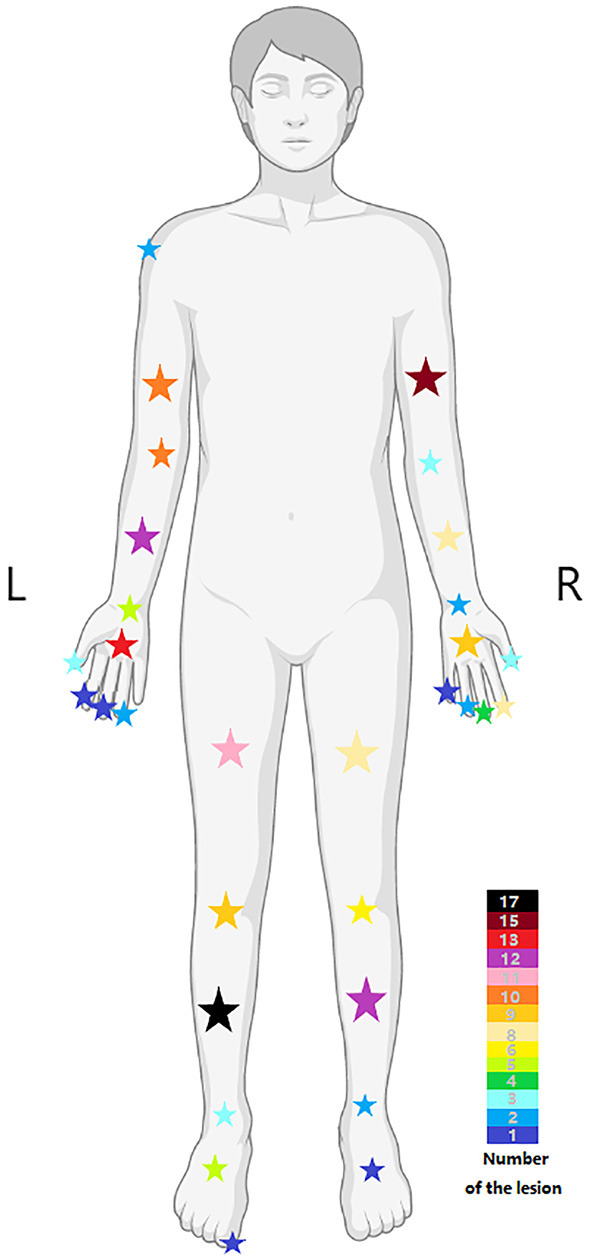
The distribution of lesions in different body regions, and the color of the stars represents the number of lesions.

In line with the currently accepted concept, isolation and singularity are the characteristics of the schwannomas in this study, with the onset peak age ranging from 20 to 60 years and no significant gender bias in the patient population (12). Similarly, the tumors often present as asymptomatic masses, and the flexor surfaces are the frequent site of schwannoma occurrence (13). However, the distribution preference of schwannomas in the upper or lower parts of the extremity is controversial. In studies discussing schwannomas of the extremity, the lower extremity tends to be more frequent (8, 9), but in retrospective studies discussing schwannomas in humans, the upper extremity tends to be more frequent (2, 14, 15). In our study, upper extremity masses were more frequent, which may result from the different definitions for the extremities. The upper extremity was defined by the shoulder, while the lower extremity was defined by the groin, and the buttock, as an intermediate region, was not included. Certainly, the variability in the characteristics of patients attending different medical centers may also contribute to the difference. Furthermore, there is a higher incidence of masses in the left extremity (55.26%), while the long bone region had the highest incidence (Figure 5).

It is generally accepted that the clinical presentation of a mass often influences whether a patient seeks medical help and that the length of medical history may likewise contribute to the manifestation of discomfort. In this study, the patients had an average medical history of about 2 years, with the longest being more than 20 years, and the large degree of dispersion led to an interest in the factors influencing the length of the disease course. The results showed that patients were more likely to notice masses in the extensor surfaces and long bone regions, and then seek early medical treatment. Besides, nearly one-third of the 114 patients with complaints of discomfort started with asymptomatic masses, and as time went by, patients gradually developed pain, numbness, tenderness, and even impaired movement, most of which appeared 1 year or more after the discovery of the masses. Therefore, early surgical removal of suspected neurogenic masses can prevent the occurrence of concomitant symptoms and even postoperative complications, thus improving the quality of patients' life (Figure 1).

Schwannomas are mostly disseminated and their most common nerve of origin is inconclusive, but the main trunk nerves, such as the median, brachial plexus, and tibial nerves, tend to have the highest incidence of masses in previous studies (6, 8, 11, 16, 17). However, we found that the digital nerve was the most commonly involved, followed by the median nerve. This reminds clinicians to consider the possibility of schwannomas in the differential diagnosis of finger masses. In addition, masses of nerve trunk origin have a higher incidence of complaints, especially numbness and tenderness, than branch nerves, and therefore surgeons need to plan better and perform careful tissue separation in patients with such complaints to minimize the possible impact on the nerve trunk. Also, unlike the findings of Abe et al. (18), there was no significant correlation between clinical symptoms and the volume of the mass in our patients. Perhaps, the volume of the tumor occurrence site may have a greater impact on the volume of the tumor (Figure 3).

### Diagnosis of schwannomas

**Figure 6:**
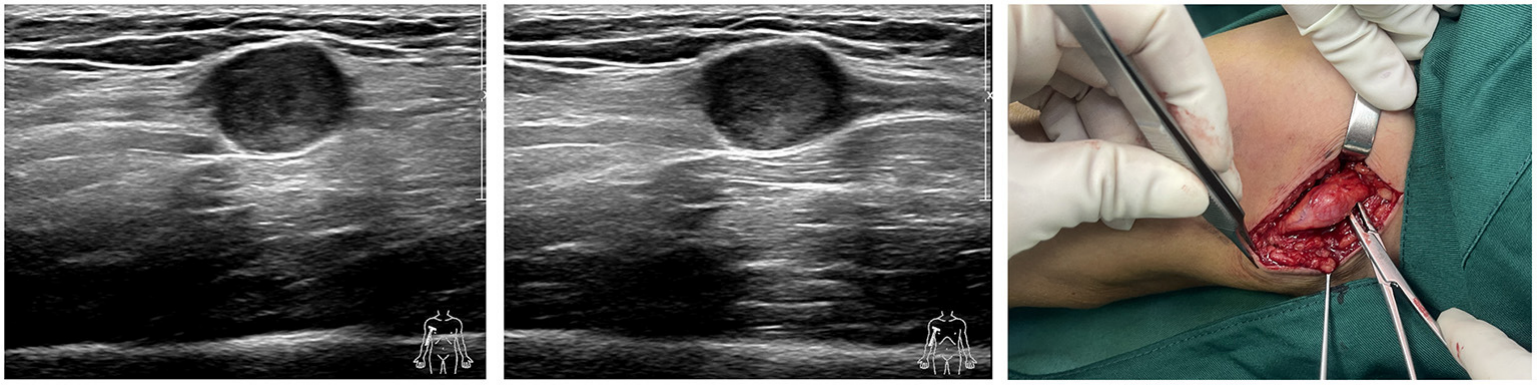
Ultrasound images and intraoperative images of schwannomas of ulnar nerve origin in the upper arm.

**Figure 7:**
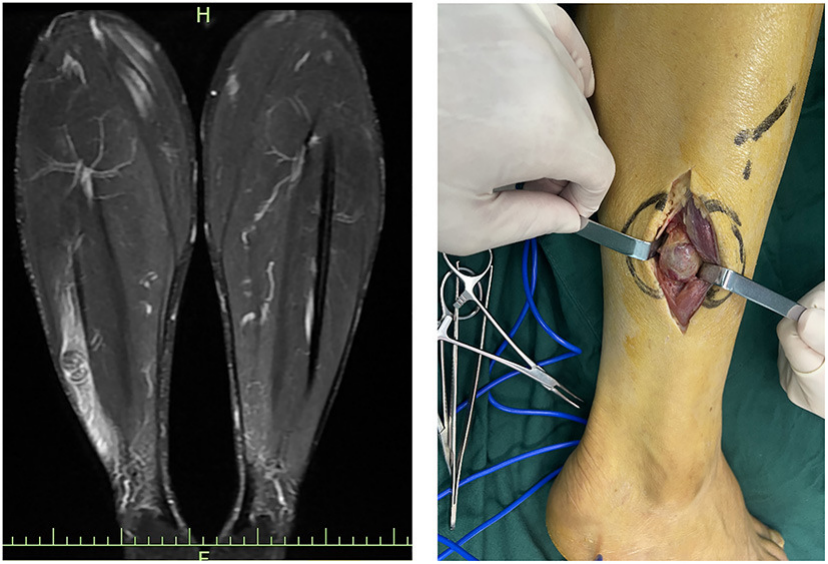
MRI shows that the tumor is located between the peroneus longus muscle.

Correct clinical diagnosis helps to better plan surgical procedures, thus reducing the possibility of intraoperative nerve injury, and the first evidence of which comes from the patient's complaints of discomfort and physical examination. However, the majority of patients presented with asymptomatic masses. Meanwhile, the knowledge of the Tinel sign varied between history-takers, and some clinicians may not even consider the Tinel sign, resulting in very limited information provided by such evidence. In this survey, only one-third of 24 patients who had not undergone imaging examination considered the possibility of a neurogenic tumor in initial diagnosis, with hemangiomas, lipomas, subcutaneous nodules, and ganglions being common considerations in the residual patients. Therefore, preoperative imaging is critical to provide abundant information about the mass, especially ultrasound and MRI. Ultrasound, with the development and application of higher resolution techniques, the characteristic mouse tail sign of schwannomas and the relationship between the mass and nerve can be better demonstrated. Although, in small peripheral nerves, such as the fingertips, the detection rate of neurogenic tumors still needs to be improved (Figure 6) (19, 20). Similar to previous studies, the characteristic target sign of MRI has a sensitivity of nearly 80%, and its eccentric position of the lesion imaging helps in the differential diagnosis of neurofibroma (8, 20). MRI also helps the surgeon to better locate, avoid possible intraoperative nerve injury, and achieve complete lesion resection (Figure 7). However, from another perspective, the fineness and complexity of MRI also limit its clinical application, due to the requirements of the higher ability of radiologists and longer review time. In our previous study, we tried to draw artificial intelligence into medical image reading and achieved a good correct rate (21). We believe that in the future, with the continuous progress of information technology, medical imaging diagnosis will enter the era of intelligence, and we are also looking forward to the feasibility of multimodal diagnosis in the field of artificial intelligence medical diagnosis, such as combining the results of multiple types of radiology, combining imaging and medical history, and combining imaging and pathology.

The definitive diagnosis of schwannomas still relies on pathology, and in the classic form, the alternating presence of Antoni A and B zones is the key to diagnosis (22). However, there are exceptions to everything. Atypical schwannomas require a combination of immunohistochemical findings to differentiate them from tumors such as neurofibromas and solitary circumscribed neuroma (23). S-100 and SOX10 are recognized positive markers, but the ancillary role of other markers, such as EMA, INI-1, and TTF-1, in the diagnosis is rarely discussed and demonstrated (24). In this study, we verified the high positive rate of S-100 and SOX in schwannomas and identified several immunohistochemical markers that could be used as potential adjunctive diagnostic markers, such as Ki-67 and β-Catenin, which had high positive rates, and SDH8, Vimentin, H3K27Me3, and Fli-1, which stained a small number of cases but achieved 100% positive rate, and perhaps they can be tested more in future immunohistochemistry to obtain more data. Interestingly, EMA markers were 100% negative in this study, which may be related to the intracapsular resection of the tumor.

### Surgical treatment and prognosis

The treatment options for schwannomas are still controversial, given the risk of medically induced nerve injury from surgery and anesthesia and incisional problems. However, with the continuous development and application of microsurgical techniques, more investigators are recommending schwannomas undergo surgical resection (25–28). In the literature, we observed two main types of complete tumor resection, intracapsular resection and extracapsular resection. Extracapsular resection ensures maximum complete removal of the tumor, and studies have reported good clinical outcomes (8, 29). However, some investigators pointed out that extracapsular resection carries the possibility of damaging the functional nerve fascicles in the capsular layer, while intracapsular resection minimizes the risk of nerve injury without a high recurrence rate (26, 30). In this study, four patients with extracapsular resection reported varying degrees of nerve injury symptoms, including simple numbness and pain, severe clawed hands, and walking discomfort, and three required reoperations. However, patients with intracapsular resection were not completely free from the risk of nerve injury, and two patients also presented with varying degrees of numbness and sensory abnormalities. All six patients had a solitary mass, and five of them were from the nerve trunk, which may be one of the risk factors for postoperative complications. Also, five patients had a medical history of more than 1 year, ranging from 20 years to 7 days. The patient with a 20-year medical history also had the largest schwannoma, about 23.56 cm^3^, but postoperatively only complained about abnormal skin sensations and tingling, which gradually vanished after 3 months. The patient with a mass history of only 7 days had the second largest schwannoma, 7.84 cm^3^, with postoperative symptoms of radial nerve entrapment and surgical release. Unlike previous studies (31, 32), tumor size, nerve origin, and the age of the patient did not show significant correlations with the occurrence of postoperative complications in the study. More data need to be collected to clarify the impact of multiple factors on postoperative complications.

**Figure 8:**
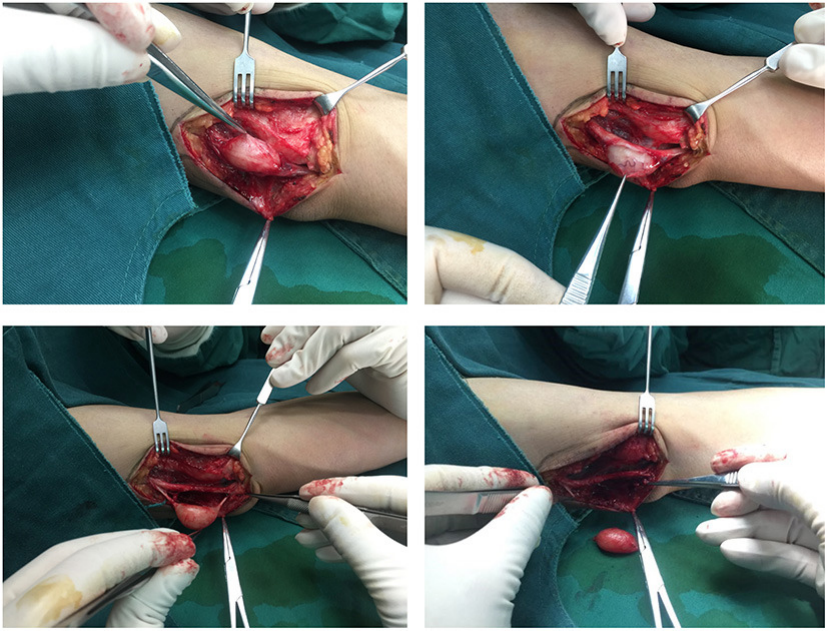
A nerve bundle was seen passing over the surface of an ulnar nerve-derived mass, which was meticulously dissected to preserve the integrity of this bundle.

**Figure 9:**
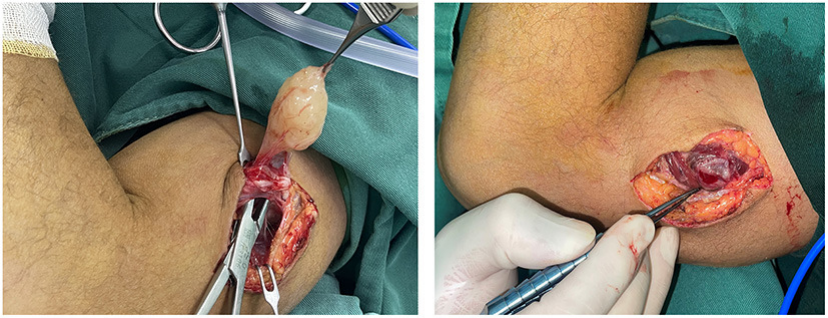
Radial nerve trunk hemorrhage after tumor shelled out.

Based on the above, we suggest that (1) meticulous microsurgical dissection should be performed for schwannomas to gain maximum preservation of nerve function; (2) sacrificing some of the nerve fiber bundles, such as the nerve fascicles that have entered the tumor, and the distal nerve fasciculus will not lead to postoperative neurological dysfunction, and to some extent, can reduce the intraoperative stimulation of the nerve trunk; (3) intracapsular resection of schwannomas located on the nerve trunk is recommended to reduce or mitigate the sequelae of nerve injury; (4) after revealing the mass and before intracapsular resection, the mass needs to be 360° exposed and searched for the presence of normal nerve fiber bundles passing through the mass, and if so, the mass is rotated vertically in the direction of the nerve trunk to find the best nerve-free zone for the incision, thus ensures complete resection of the mass and maximum preservation of the surrounding fascicles (Figure 8); (5) After shelling out the tumor, the preserved nerve should be meticulously hemostatic by electrocoagulation, or, in case of rich blood supply, local drainage and negative pressure suction are feasible to avoid nerve entrapment due to hematoma (Figure 9).

Another technical point of the operation process worth discussing is intraoperative monitoring, including nerve action potentials, compound muscle action potentials, and somatosensory evoked potentials (33). Intraoperative electrophysiological monitoring helps to clarify the anatomy and nerve orientation and to distinguish the involved nerves to maximize the preservation of healthy nerves and achieve complete tumor resection (6, 33, 34). However, there are several limitations in its clinical application. Most basically, most surgeons lack experience with the technique, and the instruments required for its implementation are absent from the operating rooms of most hospitals, such as ours (35). Second, the technique limits the choice of anesthesia; general anesthesia is required, as well as larger surgical incisions and longer operative time (33, 35, 36). This results in greater anesthetic risk, longer awakening time, and more postoperative discomfort than with routine nerve-blocking anesthesia. The larger surgical incision also increases the risk of infection and scar growth, and somewhat affects esthetics. These may explain the relatively limited number of studies exploring this technique, and more common reports of brachial plexus schwannomas and major nerve schwannomas (6, 33, 36–38), given their deeper location and greater potential of nerve invasion and complications occurrence. In addition, the lesser neural association and better prognosis of schwannomas compared with neurofibromas and malignant peripheral nerve sheath tumors may also limit its use to some extent (37). Therefore, for intraoperative electrophysiological monitoring, we recommend it for schwannomas with significant preoperative symptoms, involvement of the nerve trunk, deep sites, or large size.

Recurrence of schwannomas after surgical resection is extremely rare (6, 8, 31, 32). In this study, five patients were found to have postoperative recurrences, ranging from 1 to 20 years, and four of them had undergone tumor resection at an outside hospital before attending our hospital, thus detailed medical records are not available. It is interesting to note that in three cases, the site of recurrence was different from the primary site and recurred in the ipsilateral upper extremity or even the contralateral lower extremity.

## Limitations

The limitations of our study are inherent to its retrospective nature. Given the rarity of schwannomas, clinicians have varying degrees of awareness of such a disease, leading to possible omissions in their history-taking and examination of signs, as well as different choices of examination tools. Second, patients with schwannomatosis were not carefully screened in our study, and some patients with multiple masses may be classified as having this disease. Additionally, the description of the surgical procedure by the surgeon varies from person to person, which may have an impact on the classification of the surgical treatment. Also, the duration of follow-up varies from patient to patient, with most patients having relatively short follow-ups (Supplementary Figure 2). For the analysis of the factors influencing postoperative complications, more patients' data need to be collected to achieve this.

## Conclusion

We explored the clinical features and management of 183 cases of schwannomas in a single center, which is the largest clinical retrospective study of schwannomas in extremities. The study showed that schwannomas tend to be isolated, asymptomatic masses, occurring mostly in the left limb, flexor surface, upper extremity, and long bone regions, and are more common in middle-aged adults, without gender preference. Patients are more likely to find a mass on the extensor side and in the long bone region and have the lowest tolerance for numbness. Symptoms of the schwannomas appear progressively with time. Digital nerves are a more common origin. The discomfort of nerve trunk-originated tumor is more common. MRI has a high sensitivity for the preoperative detection of schwannomas. Early microsurgical resection of the schwannomas with meticulous hemostasis and dissection is recommended. Intracapsular resection is recommended for tumors located in the nerve trunk, and the application of intraoperative electrophysiological monitoring depends on the characteristics of the schwannomas. Surgical resection of schwannomas has few complications and rare recurrences postoperatively.

## Data availability statement

The original contributions presented in the study are included in the article/Supplementary material, further inquiries can be directed to the corresponding author.

## Ethics statement

The studies involving human participants were reviewed and approved by the First Affiliated Hospital of the College of Medicine, Zhejiang University. The patients/participants provided their written informed consent to participate in this study. Written informed consent was obtained from the individual(s) for the publication of any potentially identifiable images or data included in this article.

## Author contributions

HL designed the study. HZ, CY, and JL performed data collection. AA and YD analyzed the results. ZW, VK, MA, and SE drafted the manuscript. All authors have read and approved the final manuscript.

## Supplementary material

Supplementary Figure 1 The number of surgeons and their operation type.

Supplementary Figure 2 The time span of patients' follow-up.
