# Supplementary figures and images for: Clinical characteristics and management experience of schwannoma in extremities: Lessons learned from a 10-year retrospective study
Source: Front Neurol. 2022 Dec 15;13:1083896. doi: 10.3389/fneur.2022.1083896 (PMC9797853; doi:10.3389/fneur.2022.1083896)

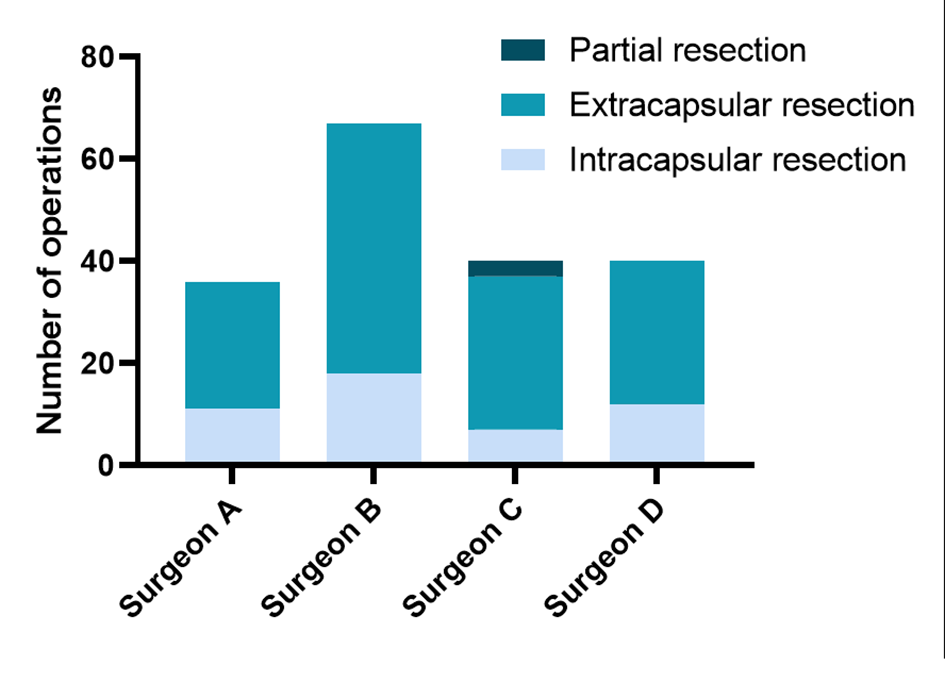

Supplement: Supplementary Figure 1 — The number of surgeons and their operation type. [file Image_1.TIF]

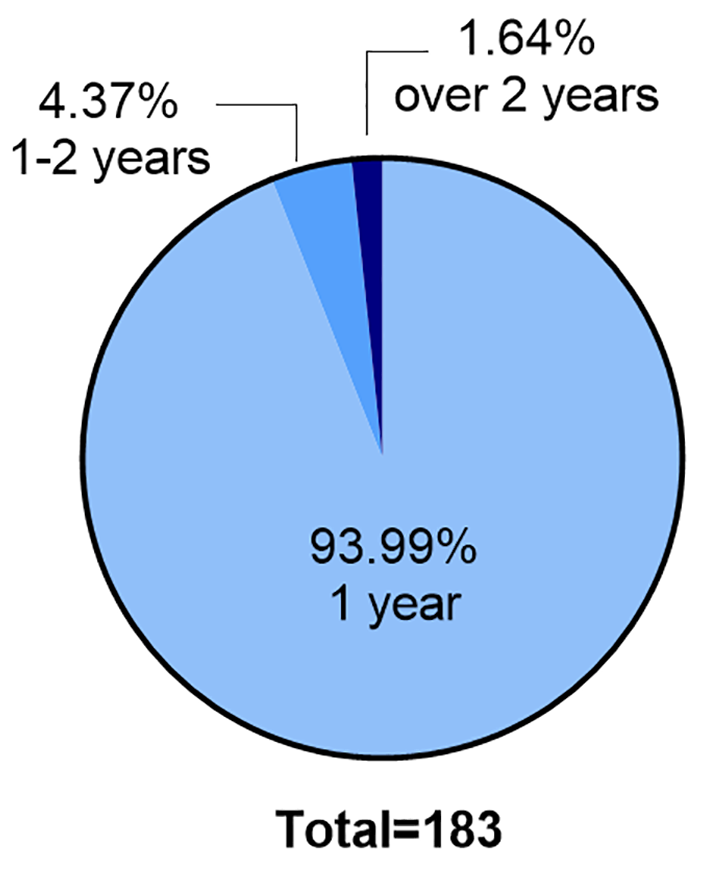

Supplement: Supplementary Figure 2 — The time span of patients' follow-up. [file Image_2.TIF]
